# Supplementary figures and images for: Spectrin coordinates cell shape and signaling essential for epidermal differentiation
Source: J Cell Biol. 2026 Feb 12;225(4):e202502071. doi: 10.1083/jcb.202502071 (PMC12898032; doi:10.1083/jcb.202502071)

Supplementary Figure 1f

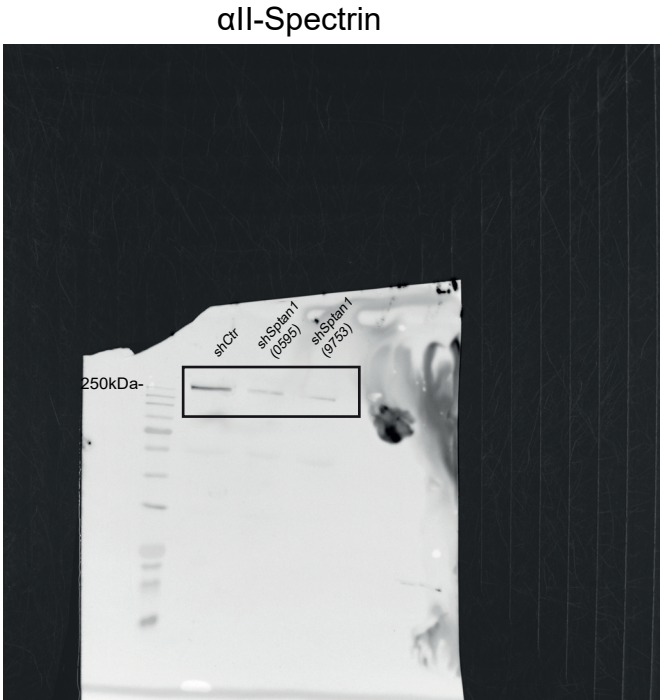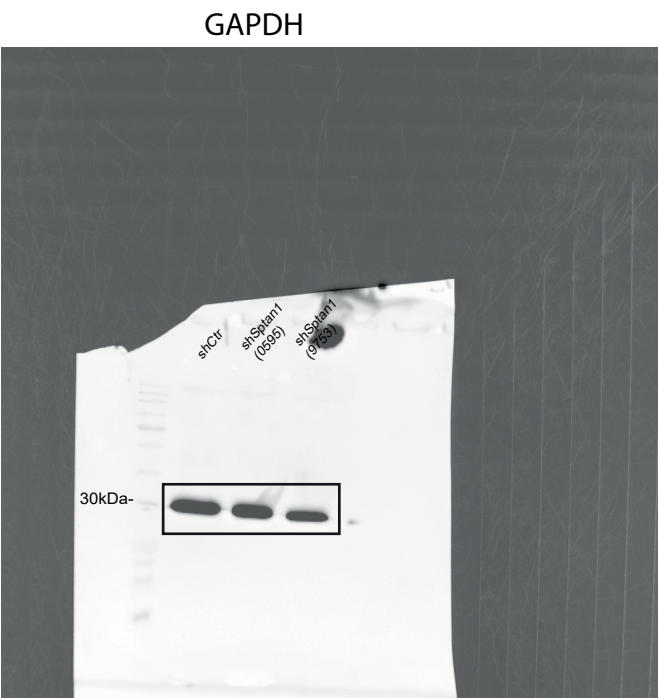

Supplementary Figure 1i

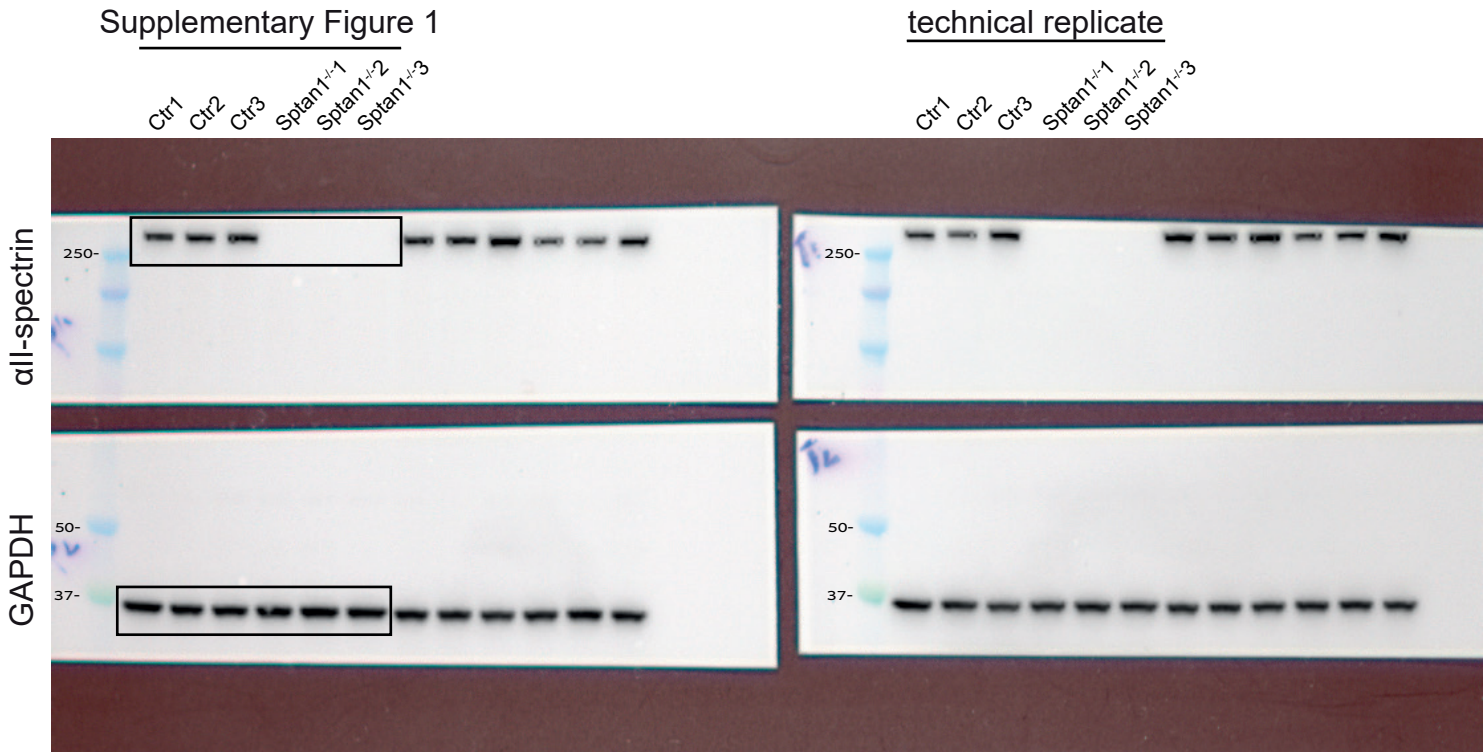

Supplement: SourceData FS1 — is the source file for Fig. S1. [file jcb_202502071_sourcedatafs1.pdf]

Supplementary Figure 2a

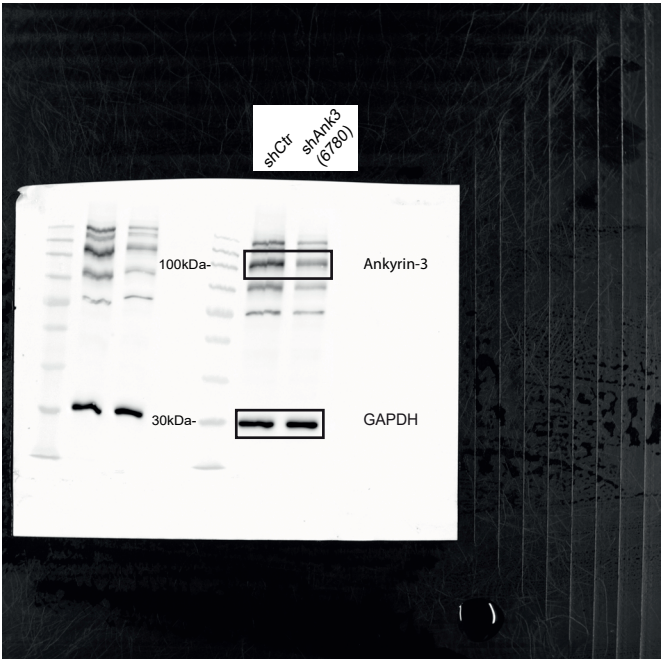

Supplement: SourceData FS2 — is the source file for Fig. S2. [file jcb_202502071_sourcedatafs2.pdf]

Supplementary Figure 3c

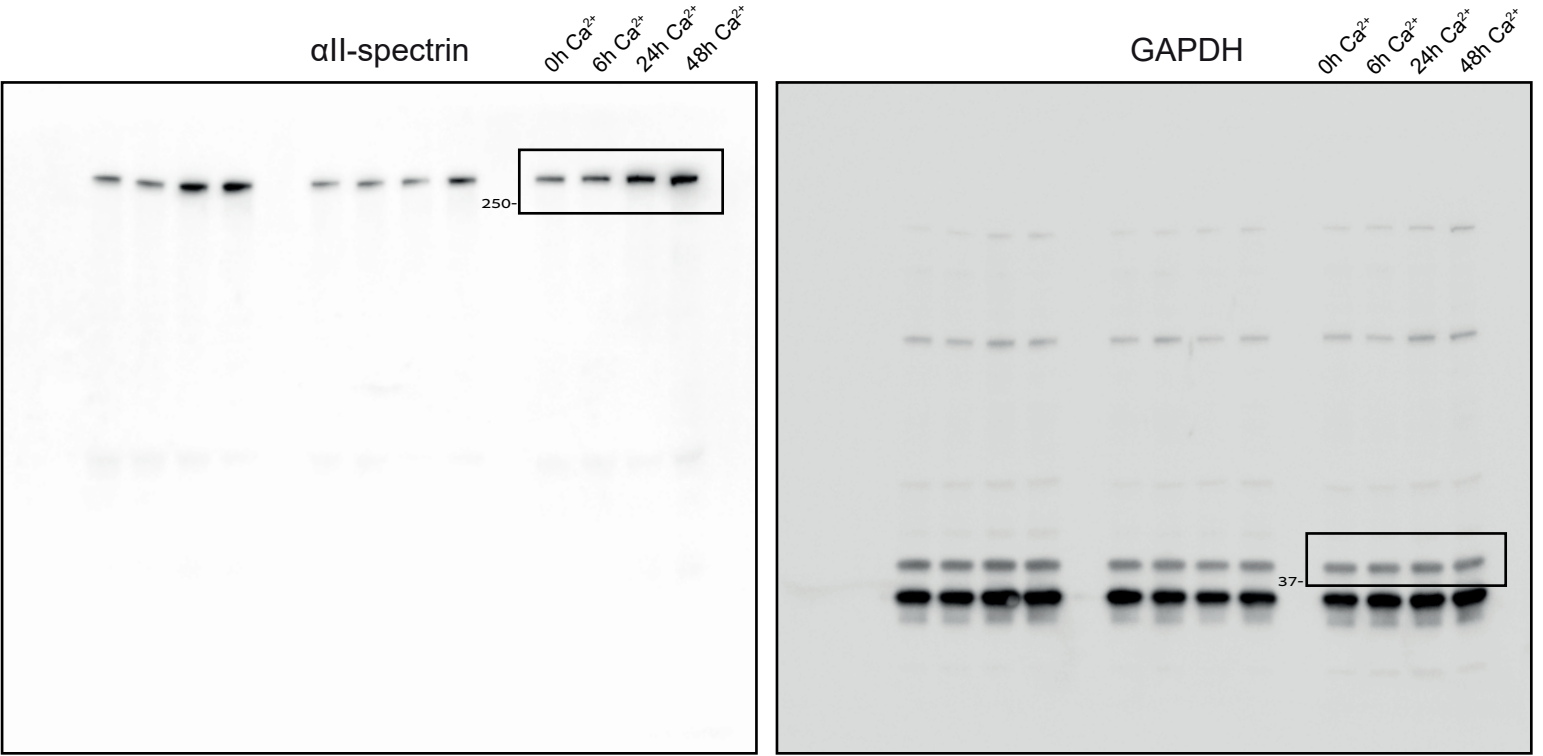

Supplementary Figure 3f

$\alpha$ II-spectrin

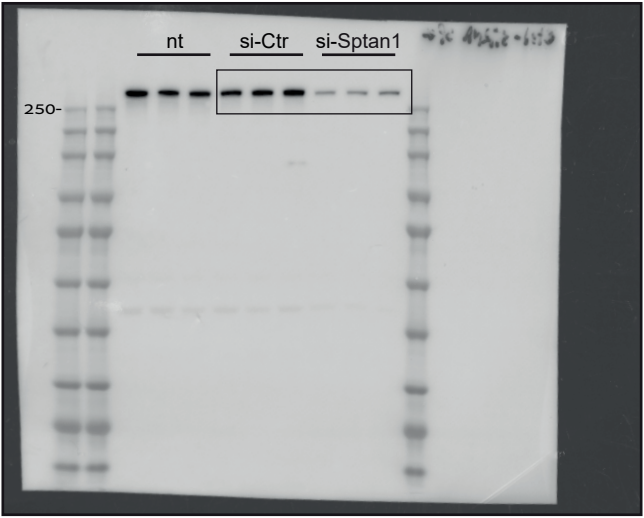

GAPDH

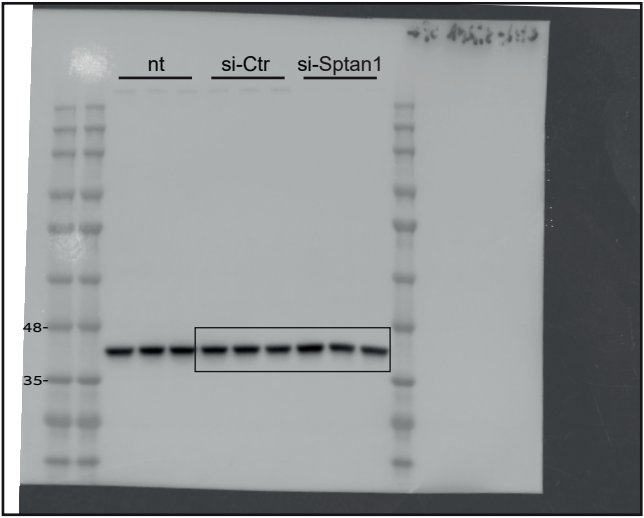

Supplement: SourceData FS3 — is the source file for Fig. S3. [file jcb_202502071_sourcedatafs3.pdf]
